# Training, Supervision, and Competence Assessment of Cameroonian Health Care Providers Using HPV Self-Sampling, Triage by Visual Inspection, and Treatment by Thermal Ablation in a Single Visit

**DOI:** 10.3389/fpubh.2022.875177

**Published:** 2022-03-28

**Authors:** Chloé Frund, Bruno Kenfack, Jessica Sormani, Ania Wisniak, Jovanny Tsuala Fouogue, Eveline Tincho, Tania Metaxas, Pierre Vassilakos, Patrick Petignat

**Affiliations:** ^1^Gynecology Division, Department of Gynecology and Obstetrics, University Hospitals of Geneva, Geneva, Switzerland; ^2^Department of Obstetrics Gynecology, Faculty of Medicine and Pharmaceutical Sciences, University of Dschang, Dschang, Cameroon; ^3^School of Health Sciences Geneva, HES-SO University of Applied Sciences and Arts Western Switzerland, Geneva, Switzerland; ^4^Faculty of Medicine and Biomedical Sciences, Centre Hospitalier Universitaire (CHUY), Yaoundé, Cameroon; ^5^Geneva Foundation for Medical Education and Research, Geneva, Switzerland

**Keywords:** cervical cancer screening, resource-limited countries, training, supervision, health care providers, visual inspection with acetic acid, thermal ablation

## Abstract

**Background:**

Developing human resource capacity and efficient deployment of skilled personnel are essential for cervical cancer screening program implementation in resource-limited countries. Our aim was to provide a context-specific training framework, supervision, and effectiveness evaluation of health care providers in a cervical cancer screening program.

**Methods:**

A 5-year cervical cancer screening program was implemented in Dschang, West Cameroon. Women were invited to perform human papillomavirus self-sampling (Self-HPV), followed by triage using visual inspection with acetic acid (VIA) and thermal ablation if needed. Health care providers were trained in four key learning phases to perform counseling, screening, and treatment process in a single visit. Training included (i) a 3-day basic course, (ii) 3-day advanced practical training, (iii) 2 weeks of supervision, and (iv) bi-monthly supervision by a mentor. The diagnostic performance of health care providers was compared between two time periods, period I (September 2018 to April 2019) and period II (May 2019 to January 2020), for an overall 17-month study period.

**Results:**

Fourteen health care providers were recruited for the training course and 12 of them completed the training objectives. Follow-up and evaluations were conducted for three health care providers working in the screening unit at Dschang District Hospital. During the study period, 1,609 women performed Self-HPV, among which 759 were screened during period I and 850 during period II. HPV positivity was 18.2 and 17.1%, and VIA positivity was 45.7 and 71.0% in period I and II, respectively. VIA sensitivity was 60.0% (95% confidence interval [CI] 26.2–87.8) and 80.8% (95% CI 60.6–93.4) in period I and II, respectively (*p* = 0.390). VIA specificity decreased between period I (57.4, 95% CI 48.1–66.3) and II (30.8, 95% CI 22.6–40.0) (*p* < 0.001). Health care providers demonstrated substantial agreement with their mentor in their diagnoses during both periods (period I: Cohen's kappa coefficient [*k*] = 0.73, 95% CI 0.62–0.85, and period II: *k* = 0.62 0.47–0.76; *p* = 0.0549).

**Discussion:**

Training, supervision, and a focus on effectiveness in cervical cancer screening are interventions that contribute to improving frontline provider competencies and maintaining a high quality of health care service delivery.

## Introduction

The burden of cervical cancer remains an important public health concern, especially in low-income countries, where it represents a leading cause of cancer death in women, despite it being a preventable disease (1, 2). In high-income countries, cytology-based programs, vaccination against high-risk human papillomaviruses (HPVs) and more recently, HPV-based screening programs, have led to an important reduction in cervical cancer incidence and mortality (3, 4). In low-income countries, these approaches have been difficult to implement, mainly owing to resource scarcity and for organizational reasons (5, 6).

In response to the problem, the World Health Organization (WHO) has launched a cervical cancer prevention initiative with the aim to eliminate cervical cancer. The WHO has defined a “90-70-90 target,” which includes (i) coverage of 90% of girls vaccinated against HPV, (ii) 70% of women screened, and (iii) 90% of women identified with cervical disease receiving treatment (7). To reach the second and third targets, the WHO recommends screening the target population with a high-performance test such as an HPV test, which may be followed by triage and prompt treatment if needed (8, 9). Currently, these procedures are relatively simple, require minimal infrastructure, and can be rapidly scaled-up if they are performed within a well-organized and structured program.

One of the constraints preventing the WHO target being met is the lack of adequately trained and qualified staff and physicians (6). In Cameroon, cervical cancer screening uptake is still very low, with only 4% of women having ever been screened, mostly owing to low awareness in the community and difficulties in accessing screening services within the public health system (6, 10, 11). The WHO has proposed decentralization and “task shifting” from physicians to other health care providers (HCPs) (e.g., midwives or nurses) to scale up cervical cancer prevention in resource-limited settings (6).

One of the first examples of innovative task shifting in the field of cervical cancer prevention was the introduction of HPV self-sampling (Self-HPV), which has been demonstrated to be as accurate as clinician sampling, suggesting that women can effectively replace health care practitioners in this process. A second example is the adoption of screen-and-treat approaches in which the treatment decision is based on a screening test, thereby avoiding colposcopy services and biopsy, which are both important barriers to cervical cancer prevention in low-resource contexts (12, 13). This allows a range of different HCPs such as physicians, nurses, and midwives to perform the screening and treatment procedures. However, to be efficient and impactful, providers should have adequate competencies and quality control of the care delivered is needed; a high level of HCP skills should also be maintained (14, 15). The approach needs to be accompanied by training, mentoring, supervision, and continuous support as well as evaluation of clinical care and patient outcomes (14). Our aim was to implement and evaluate a HCP training and supervision framework to deliver cervical cancer screening in a decentralized geographical area.

## Materials and Methods

### Setting

The screening campaign took place at Dschang District Hospital, West Cameroon between September 5, 2018 and January 10, 2020. This study was embedded in an overall research project called “3T-Approach” (for Test-Triage-and-Treat), which plans to recruit 6,000 women over a 5-year period (2018–2023) (16).

### Program for Participants

**Figure 1:**
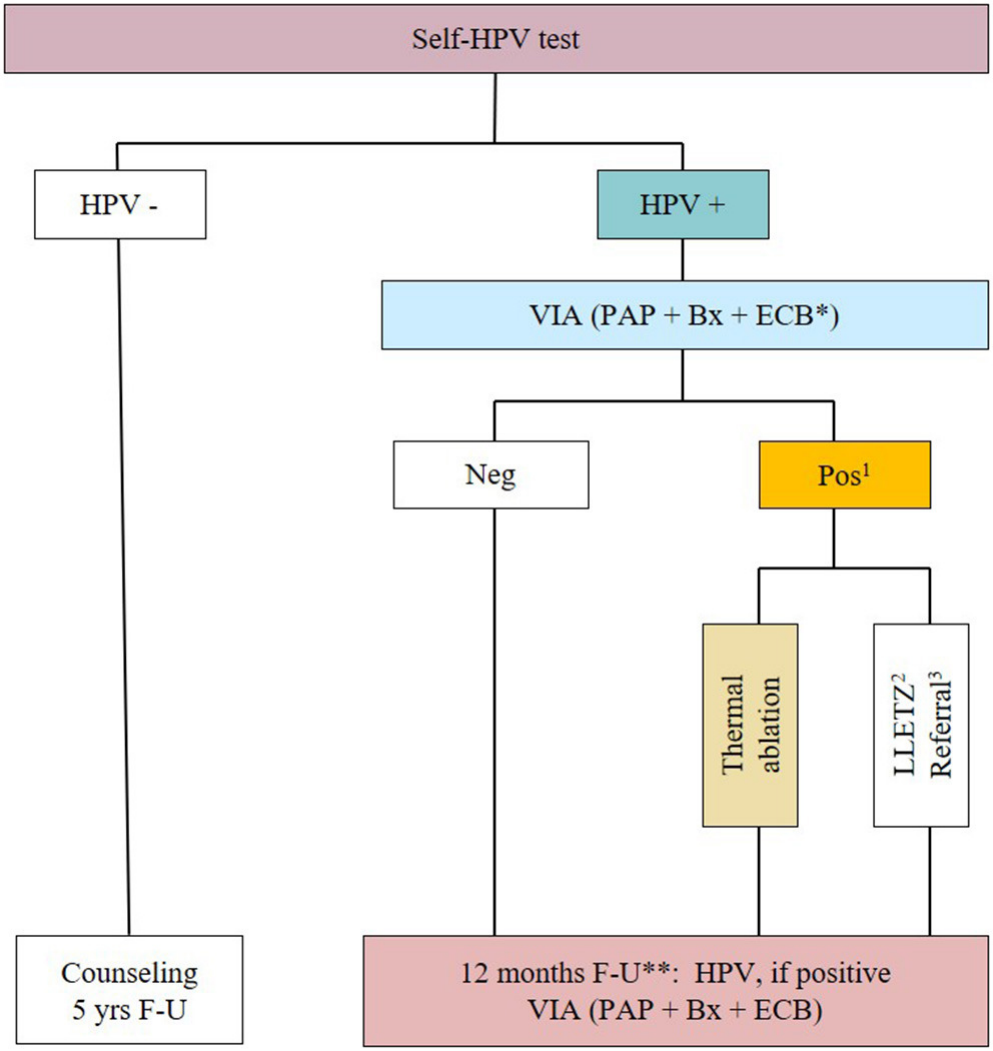
Flowchart of the study. *HPV-positive women will undergo Papanicolaou smear (PAP), cervical biopsy (Bx) and endocervical brushing (ECB); (1) Inspection for eligibility for thermal ablation; (2) If not eligible for thermal ablation; (3) If suspicious for cancer. HPV, Human papillomavirus; Self-HPV, HPV self-sampling; LLETZ, Large loop excision of the transformation zone; VIA, Visual inspection with acetic acid and Lugol's iodine; F-U: follow-up. **Women treated by thermal ablation also had a post-operative consultation at 4 to 6 weeks, and a follow-up at 6 months with Self-HPV, if positive VIA, PAP, Bx, and ECB.

The program is based on a single-day visit and consists of the following steps: (i) 1 h of community education and counseling on sexual health, HPV infection, cervical cancer prevention, and management in the case of a positive screening result; (ii) Self-HPV analyzed using a point-of-care assay (GeneXpert^®^); (iii) HPV-negative women are advised to repeat screening in 5 years; HPV-positive women are triaged using visual inspection with application of acetic acid (VIA) and Lugol's iodine (VILI) (hereafter, VIA/VILI assessment is referred to as VIA); naked-eye VIA assessment is followed by systematic digital imaging of the cervix with a smartphone (Samsung Galaxy J5, Seoul, South Korea); (iv) Papanicolaou (PAP) testing, endocervical brushing, and biopsy of VIA-positive areas or random biopsy at 6 o'clock within the transformation zone if VIA is non-pathological are conducted; and (v) treatment is provided with thermal ablation if eligible or referral for additional work-up if not eligible. VIA–positive participants eligible for ablative therapy are treated with a WiSAP thermal coagulator (Wisap^®^ Medical Technology GmbH, Brunnthal/Hofolding, Germany). Follow-up of women treated by thermal ablation or LLETZ is an HPV test at 6 and 12 months followed by VIA, PAP test, cervical biopsy and ECB (Figure 1). VIA assessment of the cervix is done according to the ABCD criteria: (A) an acetowhite area (absent on the native view and visible after acetic acid application); (B) “bleeding on touch”; (C) coloring with VILI, which may aid in confirmation or identification of small acetowhite lesions; (D) diameter >5 mm for acetowhite lesions (about the size of a pencil eraser). The ABCD criteria are positive if ACD or B are present. HCPs are instructed to treat with thermal ablation if the ABCD yield a positive result (17). To avoid endocervical bleeding, endocervical sampling for cytology and endocervical brushing is performed after VIA.

### Selection of Trainees

Fourteen HCPs participated in the training sessions. For the study setting, the hospital administration and local authorities participated in selection of the HCPs (nurses and midwives), who agreed that they would remain available to participate in the screening activity for a 5-year period. Nurses and midwives were chosen because they are trusted by women in the community and already have skills in pelvic examination.

### Three-Day On-Site Training, Basic Course

Three days were dedicated to learning and training on cancer in general, the cause and risk factors of cervical cancer from HPV disease to the development of cancer, prevention, symptoms of cervical cancer, early detection, treatment of precancerous lesions, and treatment of cancer. After the first session, participants were assessed using a multiple-choice question test covering the content of the 3-day basic course (Table 1).

**Table 1:** Components of frontline provider training using the 3T-approach.

**Length**	**Learning objectives**	**Themes**	**Teaching strategy**	**Evaluation**
3 days	Understand development of cervical precancerous and cancerous lesions Be capable of conducting a relevant medical interview Be capable of informing patients before VIA Be capable of obtaining informed consent Be capable of informing patients after VIA Be capable of referring to a specialist	CC prevention: vaccination, screening tests, target population Anatomy of a normal cervix and physiological changes in the cervix during a woman's life Pathogenesis of CC from HPV infection to cancer Screening tests: PAP, VIA, HPV test indications and procedures TA indication, procedure, and side effects Interview with patients: do's and don'ts, informed consent Infection prevention: medical equipment, waste management, hand washing Quality control and key performance indicators	On-site course Role-playing	MCQ test
3 days	Be capable of performing a pelvic exam Be capable of obtaining samples for HPV testing and cervical cytology Adopt good practices in infection prevention Be capable of identifying the transformation zone, of examining the vagina and the cervix with acetic acid and Lugol's iodine, and choosing the site of biopsy Be capable of describing and reporting a cervical exam	VIA: application of acetic acid and Lugol's iodine, smartphone image acquisition, interpretation of normal and pathologic changes Patient information: key points about cervical cancer and plain-language explanation of information How to perform an HPV test, PAP test, biopsy, endocervical brushing, and TA GeneXpert® training and HPV test interpretation Hand washing and device sterilization	On-site course Training using mannequin for pelvic exam and smartphone image acquisition Training for TA	Role play
First 2 weeks of campaign	Observe 5 health education sessions, 10 VIA, 3 TA Perform 5 supervised health education sessions, 10 VIA, 3 TA	3T-Approach	Clinical practice under supervision	On-site feedback
Continuing mentorship	Autonomous practice of educational sessions, patient registration, HPV test, VIA, and TA	3T-Approach	On-site supervisor on request Bimonthly smartphone photo review with a mentor	On-site feedback

*VIA, visual inspection with acetic acid; CC, cervical cancer; HPV, human papillomavirus; TA, thermal ablation; 3T-Approach–Test, Triage, and Treat; PAP, Papanicolaou smear; MCQ, multiple-choice question*.

### Three-Day On-Site Training, Advanced Course

**Figure 2:**
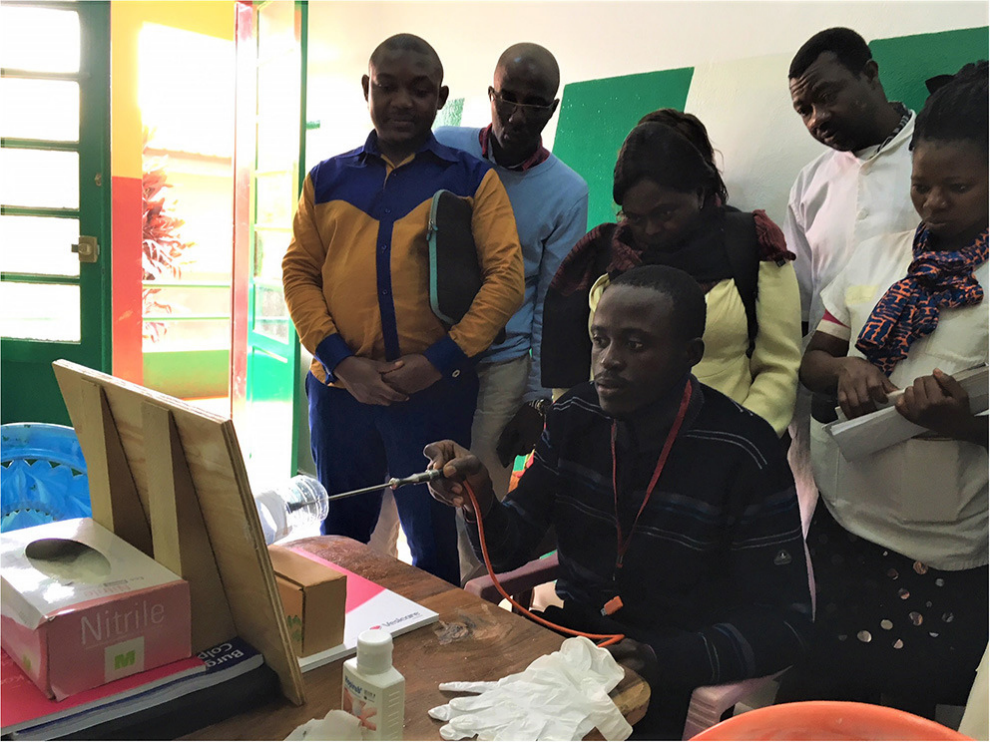
Thermal ablation simulation at Dschang district hospital. HCPs become familiar with the equipment and competent at performing thermal ablation through the use of models. Polyvinyl chloride tubes (500 cc water bottle) with a diameter of approximately 5 cm were used to simulate the vaginal canal and potatoes with a diameter of 2–3 cm were used to simulate the cervix. The probe can be placed through the simulated vagina and applied to the simulated cervix. This type of model is widely accessible and useful in achieving clinical competence before performing the procedure on a patient.

The second 3-day session was conducted 2 months later and consisted of a refresher in theoretical knowledge and advanced practical training. The trainees conducted simulated gynecologic examinations using a plastic pelvis and learned how to clean the cervix, understand the mechanics and subtleties of cervical photography, and they conducted a simulated thermal ablation procedure using potatoes. The second session was assessed via scenario-based role playing in which participants carried out simulated consultations. Various themes were covered, such as welcoming patients for their screening, counseling, explanation of the screening process, conducting VIA, description of a VIA-positive image, and informing of a precancerous lesion and recommended care (Table 1 and Figure 2).

### Two-Week On-Site Supervision

The third part of our training framework took place 2 months later when the screening campaign started and consisted of clinical practice. Didactic training was considered too brief to result in sustained changes in clinical practice; therefore, each provider first observed five patient educational sessions, 10 VIA, and three thermal ablations before supervising a minimum of five patient educational sessions, 10 VIA, and three thermal ablations with simultaneous feedback. A mentor observed HCPs' clinical skills and assessed competencies in counseling, pelvic examination and VIA procedures, smartphone image acquisition, sampling, thermal ablation, follow-up processes, and the quality of data collection.

### Activity Monitoring

A personal logbook for each HCP was used as a tool for monitoring midwifery practice. The first part of the logbook included a list of required competencies (general knowledge, clinical skills, administrative and communication skills) and the date on which the skill was acquired. In the second part of the logbook, HCPs recorded their first 100 cervical examinations performed along with the results of VIA; whether a PAP test, biopsy, and ECB was performed; whether the examination was done under supervision or autonomously; and their self-assessment of the full procedure. The last logbook was completed on January 10, 2020. Moreover, providers had to document findings on the appropriate data management forms in the health facility files as well as a case report form.

### Bimonthly Mentorship Session

Digital images (native, VIA, and VILI) were reviewed, and the HCP's diagnosis at the time of screening was discussed by the whole team and supervised by a Cameroonian gynecologist trained and experienced in VIA. During the sessions, for each HPV positive case, the digital images were uploaded to a computer. The mentor asked the HCPs to discuss why each case was considered either positive or negative. The mentor then gave their diagnosis based on the digital images. The mentor diagnoses were recorded as well as cases where photos of the cervix were not interpretable. When the mentor thought that a positive case was missed by the HCP, he used his clinical judgment to decide whether to wait for the pathology results or to recall the patient for treatment without waiting for the pathology results.

### Statistical Analysis

Cases included in the analyses were the first 100 HPV-positive cases screened by HCPs during the period under study. Two time periods were compared, period I (September 2018 to April 2019) and period II (May 2019 to January 2020) for an overall study period of 17 months. Analyses were performed using Stata Statistical Software: Release 16 (StataCorp LLC, College Station, TX, USA). Categorical variables were analyzed with Pearson's *x*^2^ or Fisher's test, as appropriate. *p*-values < 0.05 were considered significant. The sensitivity and specificity of VIA were calculated using the histologic result as reference standard, and *p*-values for sensitivity and specificity were estimated using the Z-test. The proportion of disagreement (undertreatment and overtreatment) between HCPs and mentor, using the digital VIA (DVIA) diagnosis of the mentor as a reference standard, was compared between period I and II, and *p*-values were calculated using the Z-test. Cohen's kappa coefficient (*k*) was used to measure interobserver reliability in VIA/DVIA assessment. The overtreatment rate was assessed using mentor diagnosis as the reference standard in order to replicate a real-life “screen-and-treat” approach without the availability of histological results.

## Results

### Health Care Providers

A total of 14 HCPs were recruited for the training course; 12 of them fulfilled the training objectives (Table 1), which were attendance to at least 80% of the training course and passing the theoretical and practical exams. HCPs participating in the training program were mainly nurses, midwives, and auxiliary nurses, as well as physicians and a laboratory assistant. The median age of participating HCPs was 38.3 years (interquartile range [IQR] 27–46 and the median average work experience was 9 years (IQR 4–16) (data missing for one HCP). Three HCPs with a background in midwifery were retained for the 5-year screening campaign using the 3T-Approach (Test-Triage-Treat) at Dschang District Hospital.

### Monitoring Activities

**Figure 3:**
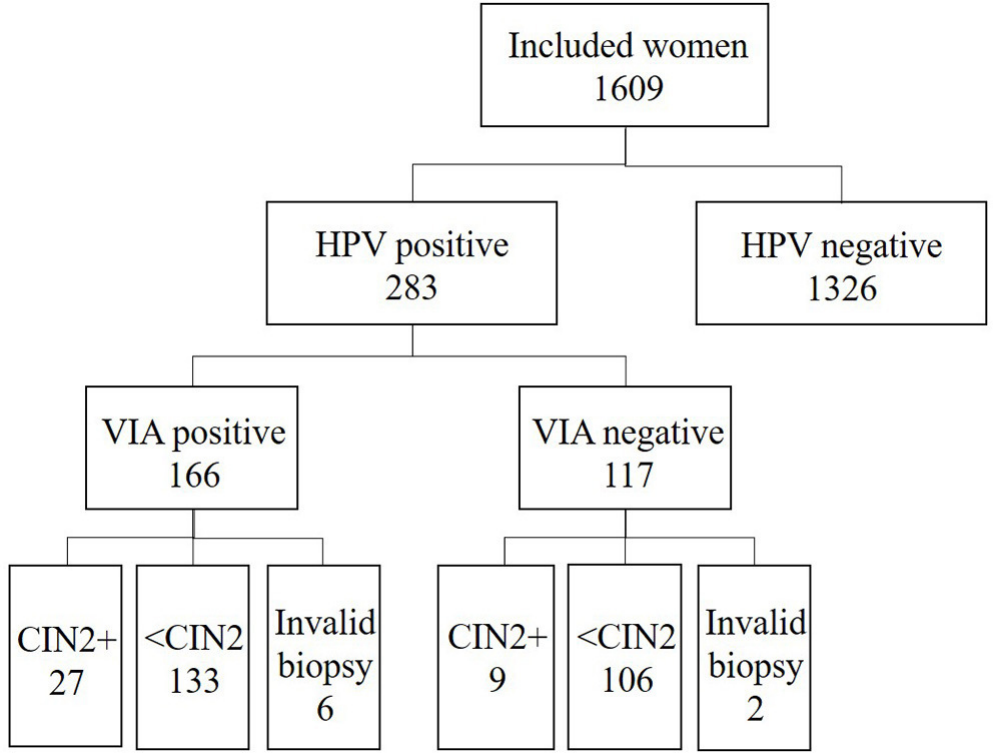
Flowchart of the included population and screening results. HPV, human papillomavirus; VIA, visual inspection with acetic acid; CIN, cervical intraepithelial neoplasia.

A total of 1,609 women were screened during the study period and the results of 300 consecutive patients with a positive HPV test result (100 patients per HCP) were selected for analysis. After exclusion of non-interpretable cases by VIA (distorted cervix or visual obstruction of the cervix [e.g., large Nabothian cysts, bleeding, or mucus], 283 cases were considered for evaluation. Overall, each HCP performed a similar number of VIA procedures. Small differences in recruitment were owing to holidays, pregnancy in one HCP, and other logistical constraints (Table 2 and Figure 3). No differences in sociodemographic characteristics of the patients examined by the HCPs were noted. We observed an increase of 25.3% in the VIA positivity rate for all HCPs combined between period I and II (45.7, 95% CI 37.2–54.3 vs. 71.0, 95% CI 62.8–78.1, *p*-value < 0.001) (Table 3). During the second period under study, we had a higher VIA positivity rate (71.0%) than the expected range between 45 and 55% (18). A larger recruitment of patients is needed to assess more precisely the VIA positivity rate. Eight patients had invalid histological results and were excluded in the calculation of sensitivity and specificity. Overall, VIA sensitivity was 60.0% (95% CI 26.2–87.8) in period I and increased to 80.8% (95 CI 60.6–93.4) in period II, but the difference did not reach statistical significance (*p* = 0.390). VIA specificity decreased between period I (57.4, 95% CI 48.1–66.3) and period II (30.8, 95% CI 22.6–40.0) (*p*-value < 0.001). Three patients were referred for suspected cervical cancer, among which one was a false positive result, one had cervical intraepithelial neoplasia grade 3, and one was confirmed as having invasive adenocarcinoma on final histological analysis. Two invasive cervical cancers were missed by the screening team upon visual assessment. One was an adenocarcinoma which was essentially endocervical and the other was an early stage, poorly differentiated carcinoma, which was interpreted as negative by the provider, although retrospective analysis of this case based on digital picture assessment has the criteria for a positive VIA. These two cases were used for continuing training of the HCP.

**Table 2:** Sociodemographic characteristics of HPV-positive participants seen by HCPs.

**Variable**	**HCP 1**	**HCP 2**	**HCP 3**	***p*** **-value**
Positive HPV test	93	95	95	
Age (y), mean±SD	38.8 (±6.2)	40.3 (±5.8)	39.3 (±6.5)	0.216
∙ Marital status				0.516
Married/in relationship Single/divorced/widowed	70 (75.3%) 23 (24.7%)	74 (77.9%) 21 (22.1%)	78 (82.1%) 17 (17.9%)	
Age at menarche (y), mean ± SD	14.8 (±2.0)	14.5 (±1.9)	14.8 (±1.7)	0.409
Age at first intercourse (y), mean ± SD	18.0 (±2.9)	18.2 (±2.7)	17.6 (±2.5)	0.318
Number of sexual partners, mean ± SD	4.3 (±3.1)	3.8 (±2.9)	4.4 (±3.9)	0.316
∙ Gravidity				0.626^*^
Nulligravida	3 (3.2%)	1 (1.1%)	1 (1.1%)	
1–5	48 (51.6%)	42 (44.7%)	48 (51.1%)	
>5	42 (45.2%)	51 (54.3%)	45 (47.9%)	
Age at first delivery (y), mean ± SD	21.2 (±5.9)	21.5 (±4.2)	20.3 (±5.7)	0.208
∙ Parity				0.866^*^
Nulliparous	4 (4.3%)	3 (3.2%)	5 (5.3 %)	
1–5	68 (73.1%)	66 (69.5%)	64 (67.4%)	
>5	21 (22.6%)	26 (27.4%)	26 (27.4%)	
∙ VIA/DVIA				0.914
Positive^**^	53 (57.0%)	56 (58.9%)	57 (60.0%)	
Negative	40 (43.0%)	39 (41.1%)	38 (40.0%)	
∙ Cytology				0.736
ASC-H, HSIL, AGC, cancer	10 (10.8%)	9 (9.5%)	7 (7.4%)	
NILM, borderline, LSIL	77 (82.8%)	82 (86.3%)	85 (89.5%)	
Invalid	6 (6.5%)	4 (4.2%)	3 (3.2%)	
∙ Histology				0.769
CIN2+	12 (12.9%)	12 (12.6%)	12 (12.6%)	
< CIN2	78 (83.9%)	82 (86.3%)	79 (83.2%)	
Invalid	3 (3.2%)	1 (1.1%)	4 (4.2%)	

*HCP, health care provider; CIN, cervical intraepithelial neoplasia; HPV, human papillomavirus; HSIL, high squamous intraepithelial lesion; LSIL, low squamous intraepithelial lesion; VIA, visual inspection with acetic acid; DVIA, digital visual inspection with acetic acid; SD, standard deviation; ASC-H, atypical squamous cell evocating high grade lesion; AGC, atypical glandular cells; NILM, negative for intraepithelial lesion or malignancy*.

* *p-value estimated using Fisher's exact test*.

** *Cases with initial suspicion of cancer are included (n = 3)*.

**Table 3:** VIA sensitivity and specificity in screening to detect CIN2+ performed by HCPs.

	**Period I (September 2018 to April 2019)**				**Period II (May 2019 to January 2020)**				
	HCP 1	HCP 2	HCP 3	**Overall**	HCP 1	HCP 2	HCP 3	**Overall**	p-value^**^
HPV positive (*n*)^*^	44	48	40	**132**	46	46	51	**143**	
CIN2+ (*n*)	3	4	3	**10**	9	8	9	**26**	
VIA/DVIA positivity rate ^***^ (95% CI)	44.7% (30.5–59.8)	43.8% (29.8–58.7)	48.8% (33.6–64.3)	**45.7% (37.2–54.3)**	69.6% (54.1–81.8)	74.5% (59.4–85.6)	69.2% (54.7–80.9)	**71.0% (62.8–78.1)**	**<0.001**
VIA/DVIA sensitivity (95% CI)	33.3% (0.8–90.6)	75.0% (19.4–99.4)	66.7% (9.4–99.2)	**60.0% (26.2–87.8)**	77.8% (40.0–97.2)	100.0% (63.1–100)	66.7% (29.9–92.5)	**80.8% (60.6–93.4)**	**0.390**
VIA/DVIA specificity (95% CI)	56.1% (39.7–71.5)	59.1% (43.2–73.7)	56.8% (39.5–72.9)	**57.4% (48.1–66.3)**	32.4% (18.0–49.8)	31.6% (17.5–48.7)	28.6% (15.7–44.6)	**30.8% (22.6–40.0)**	**<0.001**

*HPV, human papillomavirus; VIA, visual inspection with acetic acid; CIN, cervical intraepithelial neoplasia; HCP, health care provider; DVIA, digital visual Inspection with acetic acid; CI, confidence interval*.

* *With valid biopsy results (n = 275)*.

** *p-value for overall sensitivity and specificity between periods I and II*.

*** *Including all 283 patients*.

*Bold values indicate overall results (compared to the individual results of the HCPs) which are used to calculate the p-value*.

### Supervision

**Figure 4:**
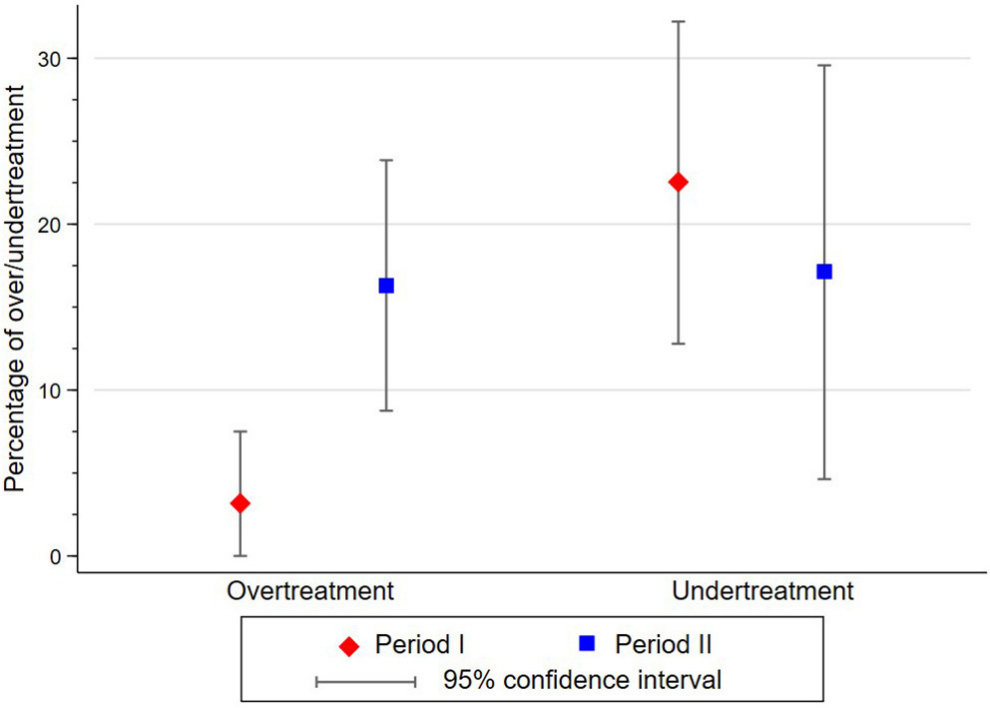
Difference in percentage of cases undertreated and overtreated by HCPs according to mentor diagnosis between period I and period II.

*S*martphone images were reviewed on a bimonthly basis. Among 283 cases photographed, 267 (94.3%) were discussed (135 in period I and 132 in period II), of which 6 (2.2%) cervical images were considered uninterpretable. Mentor review was not conducted in 16 (5.7%) cases. During period I, the HCP and the mentor agreed in 61 positive and 55 negative cases. During period II, the provider and mentor agreed in 77 positive and 29 negative cases. The proportion of agreement between HCP and mentor was 85.09% during period I and 80.3% during period II (Table 4). Cohen's kappa coefficient between HCPs and mentor over period I (*k* = 0.73, 95% CI 0.62–0-85) and period II (*k* = 0.62, 95% CI 0.47–0.76) (*p* = 0.0549) demonstrated substantial agreement. During period I, compared with the mentor diagnosis, treatment was missed by the HCP in 16 patients (22.5% of all patients diagnosed as negative by the HCP; 95% CI 12.8–32.2), and two patients underwent unnecessary thermal ablation (3.2% of all patients diagnosed as positive by the HCP; 95% CI 0.0–7.5). During period II, compared with the mentor diagnosis, treatment was missed by the HCP in six patients (17.1% of all patients diagnosed as negative by the HCP; 95% CI 4.6–29.6; *p* = 0.697 between period I and II), and 15 patients underwent unnecessary thermal ablation (16.3% of all patients diagnosed as positive by the HCP; 95% CI 8.8–23.9; *p* = 0.021 between period I and II) (Figure 4).

**Table 4:** Interobserver agreement on DVIA evaluation between HCPs and mentor.

	Period I (*n* = 135^**^; Months 0–8)	Period II (*n* = 132^**^; Months 9–17)	***p*** **-value**
**DVIA diagnosed by mentor**			
Positive	77	83	
Negative	57	44	
Non-interpretable	1	5	
Agreement on positive cases^*^	61	77	
Agreement on negative cases	55	29	
**Cohen's kappa (95% CI)**	0.73 (CI 0.62–0.85)	0.62 (0.47–0.76)	0.0549

* *Cases of initial suspicion of cancer are included (n = 3)*.

** *Cases with missing mentor diagnosis not included*.

*HCP, health care provider; CI, confidence interval; DVIA, digital visual inspection with acetic acid*.

### Patients Returning for Treatment

During period I, 15 participants (10.9% of HPV-positive participants) returned for treatment, nine upon request of the mentor and 6 after obtaining pathology results. During period II, 11 women (7.6% of 145 HPV-positive participants) returned for treatment, five on mentor supervision and six based on the pathology results.

## Discussion

Implementation of cervical cancer screening programs requires development of an effective training method focused on screening and treatment as well as thorough and accurate documentation of procedures using standardized terminology and data collection forms (19). Useful guidance, manuals, and training material for cervical cancer screening program implementation have been published by different agencies, including the Alliance for Cervical Cancer Prevention, International Agency for Research on Cancer, Johns Hopkins Program for International Education in Gynecology and Obstetrics, Pan American Health Organization, Program for Appropriate Technology in Health, and the Union for International Cancer Control. These represent important contributions to informing of evidence-based interventions within low-resource contexts (15). Our training framework was based on recommendations of the above agencies, with an approach based on four key learning steps (i) a 3-day basic course, (ii) a 3-day advanced practical training, (iii) 2-week supervision, and (iv) bimonthly supervision by a mentor. Our aim was to provide insight into how a cervical cancer screening program can function at the local level and how the quality of screening services can be sustained over time.

Our findings showed that with a structured and comprehensive training and supervision framework, HCPs could reach good levels of diagnostic performance based on HPV primary screening followed by VIA assessment, with substantial agreement between provider and mentor, which could be maintained over a 17-month period. Indeed, we observed a trend in favor of increased VIA sensitivity during the second study period, from 60.0 to 80.8% (*p* = 0.390) which did not reach significance, probably because of the small sample size. In contrast, a decrease in specificity and a small decrease in the agreement between HCP and mentor between the two periods were observed. The low specificity was expected in VIA assessment and use of the ABCD criteria in which any whitening after acetic acid application larger than 5 mm is considered positive. HCPs were instructed to consider such cases to be a positive screening result so as to minimize false negative results. In light of the high risk of loss to follow-up and considering the low risk of adverse events associated with thermal ablation, we consider the present approach to be an acceptable strategy for effective control of cervical cancer in this setting.

Over our study period, few patients (1%) were referred for suspicion of cervical cancer. This issue is particularly important in a low-resource setting where referral services are not readily available and the decision to refer is associated with additional exams and consequences for both the women and the health care system.

Within the first 2 weeks, a mentor was on-site to help providers consolidate and gain confidence in the skills obtained during training, after which regular training to maintain quality of care was put in place through a systematic bi-monthly review of cervical pictures with a multidisciplinary board. The success of our mentorship framework is largely based on the use of smartphone photography of the cervix during pelvic examination (20). DVIA has many advantages. First, cervical examination can be discussed immediately in the field with other HCPs, thus providing peer-to-peer learning opportunities, or images can be sent to an expert for real-time advice. Second, images taken during the cervical examination can be reviewed at a later time with colleagues and experts for continuous training, supervision, and quality control. Finally, if a treatment is missed, the patient can be recalled when advised by a specialist without waiting for pathology results.

It is generally difficult to achieve and maintain a well-trained cadre of providers who can consistently perform high-quality care in low-resource settings (21). In this context, supportive supervision can permit identification of gaps to quickly address any issues. To ensure a sustainable approach in a screening program, a quality-assurance system should be in place that is well-integrated among frontline providers and stakeholders, thereby allowing follow-up of the program and diagnostic performance over time (22). To date, there is a paucity of evidence regarding quality assurance and monitoring related to implementation of screening programs in low-income contexts (22). High-quality data, monitoring of these data, as well as documentation of patient-level indicators on a regular basis should be incorporated into on-site practices.

Task shifting to non-physician providers has been implemented and accepted in HIV care delivery to improve access to care and meet the demand for initiating antiretroviral therapy in more patients while maintaining high-quality services (23, 24). This promotes sustainability because non-physician providers are more likely to continue providing services than physicians, who frequently receive new assignments to other health facilities (25). Globally, there is a shortage of 1.1 million sexual, reproductive, maternal, newborn, and adolescent health (SRMNAH) professionals. Trained midwives alone could cover 90% of SRMNAH needs, but they currently only represent 10% of professionals in this field. Through our program, we can contribute to the WHO recommendation to “invest in high-quality education and training of midwives” and in “midwife-led improvements to SRMNAH service delivery” (26). However, this change needs to be accompanied by regulatory frameworks, supportive supervision, and monitoring (27).

Weaknesses of our study include the limited number of health care workers in the single setting assessed within our training framework, which may not be generalizable to other contexts. Another weakness is that women screened positive for HPV but with a negative VIA (normal-appearing cervix) have a single cervical biopsy at the transitional zone and endocervical brushing. Compared to random 4-quadrant biopsies, our approach may have possibly underestimated the true number of CIN2+.

Strengths of this study are the high quality of data collection as part of routine care and the training model based on histology as a reference standard for HPV-positive cases, enabling the objective measurement of diagnostic performance. Another strength is that the training program was based on recommendations of internationally recognized agencies for cervical cancer screening. The recent international focus on cervical cancer prevention may contribute to raising awareness about cervical cancer as well as increasing the demand for cervical cancer screening and treatment. Therefore, it is important to ensure that adequate training is provided so as to implement strategies that have a positive impact on health care systems and patient outcomes.

In conclusion, our study findings showed that non-physician HCPs can operate a cervical cancer screening program with appropriate training, supervision, and mentorship. Monitoring of clinical activity and patient outcomes are paramount to ensuring the sustainability of screening programs in low-resource settings.

## Data Availability Statement

The raw data supporting the conclusions of this article will be made available by the authors, without undue reservation.

## Ethics Statement

The studies involving human participants were reviewed and approved by the Cantonal Ethics Committee of Geneva, Switzerland (Commission Cantonale d'Ethique de la Recherche, CCER, N°2017-01110) and the Cameroonian National Ethics Committee for Human Health Research (N°2018/07/1083/CE/CNERSH/SP). It was registered on November 28, 2018 at ClinicalTrials.gov with identifier NCT03757299. The patients/participants provided their written informed consent to participate in this study. Written informed consent was obtained from the individual(s) for the publication of any potentially identifiable images or data included in this article.

## Author Contributions

CF, JS, PV, and PP contributed to the conception and design of the study. CF, BK, JS, JF, ET, and PV did the training and mentorship of the health care providers. TM wrote the logbook. CF organized the database and wrote the first draft of the manuscript. CF, JS, and AW performed the statistical analysis. CF, JS, AW and PP wrote sections of the manuscript. All authors contributed to manuscript revision, read, and approved the submitted version.

## Funding

This research was funded by ESTHER Switzerland 17G1, Service for international solidarity (State of Geneva), the University Hospital of Geneva (HUG) (Geneva, Switzerland), the Private Foundation of HUG (Geneva, Switzerland), and the Groupement Romand de la Société Suisse de Gynécologie et Obstétrique (GRSSGO) (Geneva, Switzerland).

## Conflict of Interest

The authors declare that the research was conducted in the absence of any commercial or financial relationships that could be construed as a potential conflict of interest.

## Publisher's Note

All claims expressed in this article are solely those of the authors and do not necessarily represent those of their affiliated organizations, or those of the publisher, the editors and the reviewers. Any product that may be evaluated in this article, or claim that may be made by its manufacturer, is not guaranteed or endorsed by the publisher.
